# SURGICAL TREATMENT IN CLINICAL STAGE IV GASTRIC CANCER: A COMPARISON OF DIFFERENT PROCEDURES AND SURVIVAL OUTCOMES

**DOI:** 10.1590/0102-672020210002e1648

**Published:** 2022-06-17

**Authors:** Marcus Fernando Kodama Pertille RAMOS, Marina Alessandra PEREIRA, André Roncon DIAS, Tiago Biachi de CASTRIA, Erica SAKAMOTO, Ulysses RIBEIRO-JR, Bruno ZILBERSTEIN, Sérgio Carlos NAHAS

**Affiliations:** 1 Cancer Institute, Hospital de Clínicas, Gastroenterology Department, Faculty of Medicine, University of Sao Paulo, Sao Paulo, SP, Brazil

**Keywords:** Stomach neoplasms, Gastrectomy, Neoplasm metastasis, Gastric bypass, Jejunostomy, Neoplasias gástricas, Gastrectomia, Metástase neoplásica, Derivação gástrica, Jejunostomia

## Abstract

Even in clinical stage IV gastric cancer (GC), surgical procedures may be required to palliate symptoms or in an attempt to improve survival. However, the limited survival of these patients raises doubts about who really had benefits from it.

**AIM:**

This study aimed to analyze the surgical outcomes in stage IV GC treated with surgical procedures without curative intent.

**METHODS:**

Retrospective analyses of patients with stage IV GC submitted to surgical procedures including tumor resection, bypass, jejunostomy, and diagnostic laparoscopy were performed. Patients with GC undergoing curative gastrectomy served as the comparison group.

**RESULTS:**

Surgical procedures in clinical stage IV were performed in 363 patients. Compared to curative surgery (680 patients), stage IV patients had a higher rate of comorbidities and ASA III/IV classification. The surgical procedures that were performed included 107 (29.4%) bypass procedures (partitioning/gastrojejunal anastomosis), 85 (23.4%) jejunostomies, 76 (20.9%) resections, and 76 (20.9%) diagnostic laparoscopies. Regarding patients’ characteristics, resected patients had more distant metastasis (p=0.011), bypass patients were associated with disease in more than one site (p<0.001), and laparoscopy patients had more peritoneal metastasis (p<0.001). According to the type of surgery, the median overall survival was as follows: resection (13.6 months), bypass (7.8 months), jejunostomy (2.7 months), and diagnostic (7.8 months, p<0.001). On multivariate analysis, low albumin levels, in case of more than one site of disease, jejunostomy, and laparoscopy, were associated with worse survival.

**CONCLUSION:**

Stage IV resected cases have better survival, while patients submitted to jejunostomy and diagnostic laparoscopy had the worst results. The proper identification of patients who would benefit from surgical resection may improve survival and avoid futile procedures.

## RESUMO - RACIONAL

Mesmo no câncer gástrico (CG) em estágio clínico IV (ECIV), procedimentos cirúrgicos podem ser necessários para aliviar sintomas ou na tentativa de melhorar a sobrevida. No entanto, a sobrevida limitada desses pacientes levanta dúvidas sobre quem realmente se beneficiaria.

**OBJETIVO:**

Analisar os resultados cirúrgicos do CG ECIV tratado com procedimentos cirúrgicos sem intenção curativa.

**MÉTODOS:**

Análise retrospectiva dos pacientes com CG ECIV submetido a procedimentos cirúrgicos, incluindo: ressecção tumoral, *bypass*, jejunostomia e laparoscopia diagnóstica. Pacientes submetidos à gastrectomia curativa serviram como grupo de comparação.

**RESULTADOS:**

Os procedimentos cirúrgicos em ECIV foram realizados em 363 pacientes. Comparado à cirurgia curativa (680 pacientes), os pacientes em ECIV apresentaram maior taxa de comorbidades e classificação ASA III/IV. Os procedimentos cirúrgicos realizados foram: 107 (29,4%) *bypass* (partição/anastomose gastrojejunal)*,* 85 (23,4%) jejunostomias, 76 (20,9%) ressecções e 76 (20,9%) laparoscopias diagnósticas. Em relação às características dos pacientes, os ressecados apresentaram predomínio de metástases distantes (p=0,011); os de *bypass* associaram-se a doença em mais de um sítio (p<0,001); e os laparoscópicos, metástases peritoneais (p<0,001). A sobrevida global mediana de acordo com o tipo de cirurgia foi: ressecção (13,6 meses), *bypass* (7,8 meses), jejunostomia (2,7 meses) e diagnóstica (7,8 meses) (p<0,001). Na análise multivariada, níveis baixos de albumina, mais de um sítio de doença, jejunostomia e laparoscopia associaram-se a pior sobrevida.

**CONCLUSÃO:**

Pacientes em ECIV ressecados apresentam melhor sobrevida, enquanto aqueles submetidos à jejunostomia e laparoscopia diagnóstica tiveram piores resultados. A identificação adequada dos pacientes que se beneficiariam com a ressecção cirúrgica pode melhorar a sobrevida e evitar procedimentos pouco eficazes.

## INTRODUCTION

Gastric cancer (GC) is the fifth most common cancer in the world. It is estimated that more than 1 million (1,033,701) new cases of GC occurred worldwide in 2018^4^. Surgery remains the main curative treatment option, and gastrectomy with D2 lymphadenectomy is considered the standard surgical treatment for locally advanced stage^2^ ^,^ ^22^. Unfortunately, many patients at the time of diagnosis have already locally unresectable tumors or signs of systemic disease. For clinical stage IV patients, palliative chemotherapy represents the current standard of care^18^.

However, even in stage IV, surgery may still play an important role in the treatment of GC^24^. According to its indication, the procedures performed in these patients can be classified as diagnostic, palliative, cytoreductive, and even curative. Diagnostic laparoscopy is recommended before the start of neoadjuvant chemotherapy or to confirm suspected carcinomatosis that was identified during staging exams. It may be a sole procedure or performed as the initial part of other procedures.

Meanwhile, palliative procedures are indicated in the presence of symptoms such as bleeding, perforation, or obstruction. Surgery may be an option, and it involves tumor resection or only bypass surgery. In turn, cytoreductive surgery is defined as a gastrectomy performed in asymptomatic patients harboring incurable factors such as liver/peritoneal/distant metastasis. The metastatic lesion is not resectable (R2), so the objective of the procedure is to delay the onset of symptoms by reducing tumor volume.

Recently, conversion therapy has emerged as an alternative therapy for these patients^26^. It consists of the administration of chemotherapy followed by surgery with complete resection of the tumor and associated lesions (R0). This option can be indicated to treat patients with unresectable or marginally resectable lesions, distant lymph node metastasis (LNM), and even metastatic disease or peritoneal dissemination which is still under investigation^3^.

Despite the significant number of stage IV GC, there are few reports concerning the influence of clinicopathological and treatment variables on the outcome of these patients, since the surgical series are usually focused on patients with curative resection. Accordingly, improving survival and quality of life, in contrast to the morbidity and mortality rates in these cases, remains doubtful. Thus, this study aimed to analyze the surgical results of patients with stage IV GC who underwent surgical procedures without curative intent at our institution.

## METHODS

All patients with GC, who underwent any surgical procedure from 2009 to 2020, were retrospectively evaluated in our prospective medical database. The inclusion criteria were as follows: (1) unresectable tumors, (2) signs of systemic disease, (3) R2 resections, and (4) adenocarcinoma histology. Recurrent tumor, T4b GC undergoing gastrectomy with curative intent, and conversion therapy were excluded. For analysis, patients with GC who underwent gastrectomy with curative intent served as the comparison group.

Patients were assessed preoperatively through the abdominal and pelvis analysis using computed tomography, endoscopy, and laboratory tests. All patients were staged using the TNM eighth edition. Clinical performance was evaluated by the American Society of Anesthesiologists (ASA) classification^11^, and the presence of comorbidities was classified using Charlson Comorbidity Index (CCI)^6^, without the inclusion of GC as comorbidity. All cases were operated in a high-volume center by specialized surgeons. The surgical technique, extension of resection, and dissected lymph node stations were done according to the recommendations of the Japanese Gastric Cancer Association guidelines^18^.

Postoperative complications (POC) were graded according to the Clavien-Dindo’s classification^10^. Clavien III-V was defined as a major POC. Length of hospital stay and postoperative mortality at 30 and 90 days after the procedure were evaluated as other surgical outcomes.

Concerning palliative chemotherapy, based on the REAL-2 trial, our institution has adopted a doublet containing fluoropyrimidine (capecitabine or 5-fluorouracil) and platin (oxaliplatin or cisplatin) as the preferred systemic regimen for the first line. In some cases, irinotecan and cisplatin chemotherapy was chosen to avoid infusional pump or used in those patients with difficulty in swallowing capecitabine pills. For the second line, paclitaxel and irinotecan are feasible options based on the WJOG trial. It is noteworthy that as part of the Brazilian Public Health System, in our center, monoclonal antibodies (trastuzumab or ramucirumab) as well as immunotherapy are not usually available for GC treatment^9^ ^,^ ^16^ ^,^ ^17^.

Postoperative medical appointments schedule was performed every quarter during the year or in shorter periods if necessary. Absence in appointments for more than 12 months was considered as a loss of follow-up.

This study was approved by the Hospital Ethics Committee (NP1681/20) and the National Ethics Board (CAAE: 31626220.8.0000.0068).

### Statistical analysis

The chi-square test was used to evaluate categorical variables and Student’s t-test for continuous variables. Survival was estimated using the method of Kaplan-Meier, and differences between survival curves were examined using the log-rank test. Overall survival (OS), in months, was calculated from the date of surgery until the date of death or the last contact. The factors related to 90-day mortality were analyzed by binary logistic regression analysis, and odds ratios (ORs) with 95% confidence interval (95%CI) were calculated. The Cox proportional hazards model was used to define prognostic factors related to survival. Covariates with p-values <0.05 were selected for the multivariate model. All tests were two-sided, and p-values <0.05 were considered statistically significant. The analysis was performed using SPSS software, version 20.0 (SPSS Inc, Chicago, IL).

## RESULTS

**Figure 1 -:**
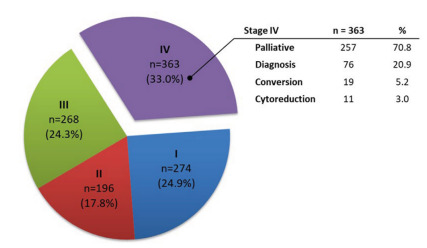
Distribution of patients with gastric cancer, according to clinical stage.

During the study period, 1188 patients with GC underwent surgical procedure at our Institution. Of these, 87 were excluded due to non-adenocarcinoma histology, and the remaining 1101 cases were included in the initial analysis. Surgical procedures were performed in 363 (33%) clinical stage IV patients (Figure 1). The indication of surgical procedure in the remaining patients with stage IV GC was performed to palliate symptoms in 257 (70.8%) cases followed by diagnosis in 76 (20.9%) cases and cytoreduction in 11 (3%) cases. Conversion surgery was performed in 19 (5.2%) cases, and these patients were excluded from further analysis. Curative intent resection was performed in 680 (61.7%) patients. Thus, a total of 344 patients met the inclusion criteria, were classified as stage IV GC, and were compared to 680 patients who underwent curative gastrectomy (Table 1).

**Table 1 -:** Clinicopathological characteristics and surgical results of curative resections compared to procedures performed in clinical stage IV patients.

Variables	Curative (D1 or D2) (n=680)	Stage IV (n=344)	p-value
Sex			0.219
Female	266 (39.1)	121 (35.2)	
Male	414 (60.9)	223 (64.8)	
Age (years)			0.378
Mean (SD)	62.9 (12.6)	62.2 (13.1)	
Median (range)	64.5 (22.7-94.5)	64.6 (24-87.9)	
BMI (kg/m²)			**<0.001**
Mean (SD)	24.4 (5.0)	22.0 (4.5)	
Median (range)	24.1 (12.5-56.5)	21.7 (13.3-38)	
Charlson Comorbidity Index			**0.011**
0	447 (65.7)	253 (73.5)	
≥1	233 (34.3)	91 (26.5)	
ASA			**<0.001**
I/II	505 (74.3)	191 (55.5)	
III/IV	175 (25.7)	153 (44.5)	
Hemoglobin (g/dL)			**<0.001**
Mean (SD)	12.3 (5.2)	10.5 (2.1)	
Albumin (g/dL)			**<0.001**
Mean (SD)	4.0 (1.3)	3.5 (0.6)	
Neutrophil to lymphocyte ratio			**<0.001**
Mean (SD)	2.79 (2.66)	5.05 (5.68)	
Lauren histological type			**<0.001**
Intestinal	366 (53.8)	59 (17.2)	
Diffuse/mixed	292 (42.9)	118 (34.3)	
Undefined	22 (3.2)	167 (48.5)	
Postoperative complications (POC)			0.203
Non/minor POC	577 (84.9)	302 (87.8)	
Major POC	103 (15.1)	42 (12.2)	
Length of hospital stay (days)			**<0.001**
Mean (SD)	12.9 (10.8)	7.4 (7)	
30-day mortality			**<0.001**
No	657 (96.6)	291 (84.6)	
Yes	23 (3.4)	53 (15.4)	
90-day mortality			**<0.001**
No	629 (92.5)	238 (69.2)	
Yes	51 (7.5)	106 (30.8)	

P-values indicated in bold are statistically significant.

SD: standard deviation; ASA: American Society of Anesthesiologists; BMI: body mass index.

Stage IV patients had significantly lower BMI, albumin, and hemoglobin levels than the curative group. ASA scores were higher for stage IV GC, but Charlson’s comorbidity index was inferior compared to patients with curative GC. Almost half of the stage IV patients did not have a defined Lauren histological type. Length of hospital stay was lower in the stage IV group, while the mortality rate at 30 and 90 days was higher than in the curative group.

### Outcomes in gastric cancer stage IV

Considering only the 344 patients with stage IV GC, the most commonly performed surgical procedure was the gastric bypass, which includes gastrojejunal anastomosis or gastric partitioning with gastrojejunal anastomosis, performed in 107 (29.4%) patients. Primary tumor resection was performed in 76 (20.9%) patients. The remaining 85 (23.4%) and 76 (20.9%) patients underwent jejunostomy and diagnostic laparoscopy, respectively.

Clinical and surgical characteristics of patients with GC according to the type of surgery are demonstrated in Table 2. Patients who underwent bypass were older and had higher ASA. Resected patients had a higher BMI than other groups. Lower hemoglobin and albumin levels were related to bypass and jejunostomy patients. Peritoneal metastasis was more frequent in patients who underwent jejunostomy and diagnostic laparoscopy, while the locoregional disease was associated with bypass procedures. More than one metastatic site was frequent in the bypass group.

**Table 2 -:** Clinicopathological characteristics of clinical stage IV patients according to the type of surgery.

Variables	Resection	Bypass	Jejunostomy	Diagnostic	p-value
	n=76 (%)	n=107 (%)	n=85 (%)	n=76 (%)	
Sex					0.388
Female	25 (32.9)	32 (29.9)	33 (38.8)	31 (40.8)	
Male	51 (67.1)	75 (70.1)	52 (61.2)	45 (59.2)	
Age (years)					**0.014**
Mean (SD)	62.8 (12.1)	64.3 (11.3)	62.6 (13)	58.1 (15.6)	
BMI (kg/m²)					**0.001**
Mean (SD)	23.3 (4.7)	21.2 (4.0)	21.1 (4.0)	22.9 (4.9)	
Charlson comorbidity index					0.711
0	52 (68.4)	81 (75.7)	63 (74.1)	57 (75)	
≥1	24 (31.6)	26 (24.3)	22 (25.9)	19 (25)	
ASA					**0.045**
I/II	43 (56.6)	51 (47.7)	45 (52.9)	52 (68.4)	
III/IV	33 (43.4)	56 (52.3)	40 (47.1)	24 (31.6)	
Hemoglobin (g/dL)					**<0.001**
Mean (SD)	10.6 (2.1)	9.6 (1.8)	10.8 (2.2)	11.4 (2.1)	
Albumin (g/dL)					**0.006**
<3.5	15 (22.4)	46 (46.5)	31 (47.7)	21 (35)	
≥3.5	52 (77.6)	53 (53.5)	34 (52.3)	39 (65)	
Neutrophil to lymphocyte ratio					0.223
Mean (SD)	4.50	4.76	6.22	4.77	
Local/locoregional disease*					**<0.001**
No	44 (57.9)	21 (19.6)	43 (50.6)	62 (81.6)	
Yes	32 (42.1)	86 (80.4)	42 (49.4)	14 (18.4)	
Peritoneal metastasis					**<0.001**
No	43 (56.6)	53 (49.5)	33 (38.8)	16 (21.1)	
Yes	33 (43.4)	54 (50.5)	52 (61.2)	60 (78.9)	
Distant metastasis					**0.011**
No	57 (75)	86 (80.4)	74 (87.1)	71 (93.4)	
Yes	19 (25)	21 (19.6)	11 (12.9)	5 (6.6)	
Number of sites with disease					**<0.001**
One	68 (89.5)	62 (57.9)	67 (78.8)	73 (96.1)	
Two or more	8 (10.5)	45 (42.1)	18 (21.2)	3 (3.9)	

*Includes T4b unresectable tumors and lymph nodes metastasis.

P-values indicated in bold are statistically significant.

SD: standard deviation; ASA: American Society of Anesthesiologists; BMI: body mass index.

After the procedure, the mean length of hospital stay was longer for patients who underwent resection. There were no significant differences in the rate of major POC between the groups. However, mortality at 30 and 90 days after the procedure was higher for patients who underwent jejunostomy. During the follow-up, patients in the resection group were able to receive more lines of palliative chemotherapy (Table 3).

**Table 3 -:** Outcomes of clinical stage IV patients according to the type of surgery.

Variables	Resection	Bypass	Jejunostomy	Diagnostic	p-value
	n=76 (%)	n=107 (%)	n=85 (%)	n=76 (%)	
Postoperative complications (POC)					0.196
No/minor POC	64 (84.2)	93 (86.9)	73 (85.9)	72 (94.7)	
Major POC	12 (15.8)	14 (13.1)	12 (14.1)	4 (5.3)	
Length of hospital stay (days)					**<0.001**
Mean (SD)	12.5 (11.0)	7.6 (5.9)	6.3 (5.3)	3.3 (4.8)	
30-day mortality					**<0.001**
No	70 (92.1)	95 (88.8)	59 (69.4)	67 (88.2)	
Yes	6 (7.9)	12 (11.2)	26 (30.6)	9 (11.8)	
90-day mortality					**<0.001**
No	65 (85.5)	74 (69.2)	41 (48.2)	58 (76.3)	
Yes	11 (14.5)	33 (30.8)	44 (51.8)	18 (23.7)	
First-line palliative treatment					**0.015**
No	27 (35.5)	43 (40.2)	43 (50.6)	20 (26.3)	
Yes	49 (64.5)	64 (59.8)	42 (49.4)	56 (73.7)	
Second-line palliative treatment					**0.028**
No	53 (69.7)	80 (74.8)	69 (81.2)	46 (60.5)	
Yes	23 (30.3)	27 (25.2)	16 (18.8)	30 (39.5)	
Third-line palliative treatment					**0.027**
No	64 (84.2)	97 (90.7)	83 (97.6)	70 (92.1)	
Yes	12 (15.8)	10 (9.3)	2 (2.4)	6 (7.9)	
Palliative/hemostatic radiotherapy					0.800
No	67 (88.2)	99 92.5)	77 (90.6)	69 (90.8)	
Yes	9 (11.8)	8 (7.5)	8 (9.4)	7 (9.2)	

P-values indicated in bold are statistically significant.

SD: standard deviation.

### Survival analysis

**Figure 2 -:**
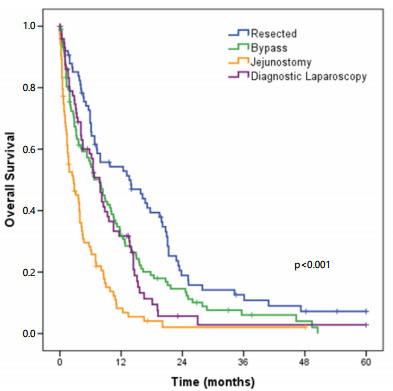
Overall survival of stage IV gastric cancer patients, according to the type of procedure.

The median OS for all patients with clinical stage IV GC was 6.2 months. According to the type of surgery, patients who underwent jejunostomy had worse survival when compared to other groups (p<0.001 for all). Also, resected patients had significantly longer survival than patients who underwent bypass, diagnostic laparoscopy, and jejunostomy (p=0.009, 0.001, and <0.001, respectively). The median OS according to the type of surgery was as follows: resection (13.6 months), bypass (7.8 months), jejunostomy (2.7 months), and diagnostic (7.8 months) (Figure 2).

In multivariate analysis, higher NLR and jejunostomy were considered independent risk factors for mortality to evaluate the characteristics associated with 90-day mortality (Table 4). Regarding OS, female gender, low albumin levels, jejunostomy, diagnostic laparoscopy, and two or more sites of disease were independent factors associated with worse OS in stage IV GC (Table 4)

**Table 4 -:** Univariate and multivariate analysis for the risk of 90-day mortality and OS.

90-day mortality	Univariate		p-value	Multivariate		p-value
Variables	OR	95%CI		OR	95%CI	
Male (vs. female)	0.90	0.56-1.46	0.675	-	-	-
Age ≥65 (vs. <65 years)	0.76	0.48-1.21	0.250	-	-	-
BMI<18.5 (vs. ≥18.5)	1.09	0.63-1.87	0.765	-	-	-
ASA III/IV (vs. I/II)	1.72	1.09-2.73	**0.021**	1.76	0.99-3.13	0.053
Charlson ≥1 (vs. 0)	1.07	0.64-1.79	0.800	-	-	-
Hb<13 (vs. ≥13)	1.39	0.65-2.95	0.395	-	-	-
Alb<3.5 (vs. ≥3.5)	2.44	1.45-4.10	**0.001**	1.77	1.00-3.13	0.050
NLR≥2.5 (vs. <2.5)	4.10	2.23-7.54	**<0.001**	2.57	1.30-5.06	**0.006**
Type of surgery (vs. resected)				-	-	-
Bypass	2.63	1.23-5.63	**0.012**	1.76	0.74-4.20	0.201
Jejunostomy	6.34	2.94-13.66	**<0.001**	5.17	2.12-12.64	**<0.001**
Diagnostic laparoscopy	1.83	0.80-4.20	0.152	1.60	0.61-4.22	0.342
**Overall survival**	**Univariate**		**p-value**	**Multivariate**		**p-value**
**Variables**	**HR**	**95%CI**		**HR**	**95%CI**	
Male (vs. female)	0.68	0.53-0.87	**0.002**	0.71	0.54-0.93	**0.014**
Age≥65 (vs. <65 years)	0.87	0.69 -1.10	0.255	-	-	-
BMI<18.5 (vs. ≥18.5)	1.04	0.78-1.37	0.799	-	-	-
ASA III/IV (vs. I/II)	1.13	0.90-1.43	0.285	-	-	-
Charlson ≥1 (vs. 0)	1.03	0.80-1.33	0.798	-	-	-
Hb<13 (vs. ≥13)	1.21	0.84-1.73	0.301	-	-	-
Alb <3.5 (vs. ≥3.5)	1.61	1.25-2.08	**<0.001**	1.55	1.17-2.05	**0.002**
NLR ≥2.5 (vs. <2.5)	1.39	1.08-1.78	**0.010**	1.30	0.99-1.72	0.063
No. of sites ≥2 (vs. 1)	1.27	0.97-1.67	0.086	1.41	1.01-1.97	**0.042**
Type of surgery (vs. resected)						
Bypass	1.52	1.10-2.09	**0.011**	1.11	0.76-1.62	0.606
Jejunostomy	3.25	2.30-4.59	**<0.001**	2.59	1.76 -3.83	**<0.001**
Diagnostic laparoscopy	1.68	1.17-2.41	**0.005**	1.58	1.06-2.37	**0.025**

P-values indicated in bold are statistically significant.

Variables with p<0.100 were included in the multivariate model.

HR: hazard ratio; ASA: American Society of Anesthesiologists; BMI: body mass index; HB: hemoglobin; Alb: albumin; NLR: neutrophil to lymphocyte ratio.

## DISCUSSION

The selection of appropriate therapy for patients with stage IV GC can be a challenge, where it is often difficult to predict if the addition of any surgical procedure to systemic chemotherapy will be beneficial. Accordingly, this population-based study evaluated the outcomes of surgical treatment on clinical stage IV GC in Western patients to provide further information to guide best clinical practices. As a result, this study demonstrated that, unfortunately, most of the patients (33%) with GC are diagnosed at this stage. These patients had poorer clinical performance, diffuse histology, and a higher rate of postoperative mortality. Still, according to the surgical modalities, we observed differences in survival between treatment approaches, where patients who underwent jejunostomy were associated with worse prognosis, while the ones who underwent resection had a significant improvement in survival rates. Furthermore, we also demonstrated that the number of metastatic sites was an independent prognostic factor related to survival.

In conformity with the TNM eighth edition, the clinical stage is determined based on the data collected about the extent of the tumor from the moment of diagnosis until the initiation of primary treatment^1^. Besides usual preoperative image studies, observations made at surgical exploration without resection are also incorporated to define the clinical stage. The clinical stage allows comparison of characteristics and outcomes of all patients with GC, including those who were not submitted to surgical resection. As expected, compared to patients treated with curative intent, stage IV GC demonstrated inferior clinical performance, evidenced by lower levels of albumin, hemoglobin, BMI, and higher ASA. Interestingly, the presence of comorbidity was inferior in patients with stage IV GC. Similar findings have been reported in other studies^12^^,^ ^13^ ^,^ ^20^ ^,^ ^23^.

In addition to the worse prognosis, many patients with stage IV GC still develop complications during the course of the disease which require palliative procedures^18^ ^,^ ^19^. One of the widest indications of surgery in stage IV GC is to palliate symptoms, such as bleeding, ascites, intestinal obstruction, and gastric outlet obstruction (GOO). The incidence of GOO is common in patients with distal GC, ranging between 5% and 14.9%^23^. Palliative resection of the tumor is the procedure of choice in cases of resectable lesions and limited metastatic disease, being an option for patients with favorable clinical conditions^15^. Indeed, there is always a concern about the morbidity and mortality of palliative resections^19^ ^,^ ^25^. In our study, the overall postoperative mortality rate within 30 days was 7.9% for resected patients. Despite the indisputable risks of surgical morbidity and prolonged hospitalization associated with palliative surgery, postoperative outcomes seem to be determined not only by the intention of the procedure exclusively, but also probably by the performance of the patients^14^. As is known, patients in good condition have better tolerance to chemotherapy and are less likely to have complications from surgical procedures^8^. And as seen in the present study, the best results of survival (13.6 months) and the highest achieved rates of administration of palliative chemotherapy corroborate the indication for resection in fit symptomatic patients^14^.

For many years, the benefit of cytoreductive surgery in asymptomatic patients is not clear. At present, after the results of the REGATTA trial, its indication has decreased^13^. In that study, asymptomatic patients with a single non-curable factor were randomized to gastrectomy followed by chemotherapy or to exclusive palliative chemotherapy. The results obtained demonstrated no survival benefit of additional gastrectomy over chemotherapy alone. Criticisms of that study remain due to the high proportion of patients with carcinomatosis, and whether there would be benefit from cytoreductive surgery in cases with tumor regression after initial cycles of palliative chemotherapy^5^ ^,^ ^7^ ^,^ ^12^. In our study, 76 patients underwent resection, which included a low frequency of cytoreductive surgeries (11 cases). These few cases were performed when patients were referred for surgery, and during the procedure, a metastasis was identified or whose surgery would be R2.

Unfortunately, due to local invasion of adjacent structures, or patients’ unfavorable clinical conditions, many of these tumors are considered unresectable at diagnosis. Surgical bypass or endoscopic stents are options to restore gastrointestinal continuity. Endoscopic stents have the advantage of being less invasive without the need for an operating room. But, in the long term, tumor growth can lead to stent obstruction with the need for reinterventions^21^ . In contrast, patients with better clinical conditions and with the possibility of receiving palliative chemotherapy have a potential benefit of definite surgical gastric bypass. The most traditional surgery performed is gastrojejunostomy (GJ). Gastric partitioning (GP) associated with GJ (also known as GP) has also been considered an option for the treatment of malignant GOO^23^. In the present study, the median OS for the bypass group was 7.8 months, and more than half of the patients received first-line palliative chemotherapy. These results were inferior to the resection group, but the higher frequency of carcinomatosis and more than one site of metastatic disease may have influenced^15^.

An interesting finding in the present cohort was related to the prognosis of jejunostomy. The indication for this procedure was the impossibility of surgical resection or internal bypass of the primary lesion. The low median survival (2.7 months) observed in our results for this group raises the question of who would benefit from this procedure. In the analysis of factors associated with 90-day mortality, which we considered an adequate period to verify whether the procedure was worthwhile, we found that jejunostomy stood out as an independent factor of poor prognosis. Furthermore, the female sex, lower albumin levels, high-NLR, and more than one metastatic site were associated with worse survival in multivariate analysis.

Although it seems a similar procedure to jejunostomy, diagnostic laparoscopy patients had no symptoms, and the procedure served only to confirm the diagnosis of stage IV disease. Even though this group had the highest frequency of peritoneal metastases, a known factor of poor prognosis, they had no obstructive lesion at the time of the procedure. This fact enabled 73.7% of the patients to receive first-line palliative chemotherapy, which comprised the higher proportion among all groups.

Some limitations of the current study should be addressed. First, we only explored patients who performed some surgical procedures. Patients with GC treated exclusively with palliative chemotherapy were not included. Thus, the incidence of stage IV GC may be even greater than reported. Second, obstructed patients who underwent palliative stenting were not included. This modality is reserved for cases of high surgical risk, with an expected survival of less than 2 months. Finally, although many of our patients received systemic chemotherapy, no single regimen was uniformly employed.

Despite these limitations, the study includes a well-characterized cohort of patients with GC treated at a referral center, where patients were evaluated and the results were compared according to the main surgical approaches in the palliative context. Furthermore, the diagnostic laparoscopy group may serve as a control for patients treated exclusively with palliative chemotherapy who had a low incidence of distant metastatic disease and who, for this reason, underwent diagnostic laparoscopy for further investigation of peritoneal disease.

In summary, the benefits of the surgical approach for stage IV GC are still uncertain in some patients with poor performance and more than one site of metastasis. However, our findings suggested that surgical resection may still play an important role in selected patients. Appropriate criteria for selected patients who could benefit have yet to be identified in order to establish the best therapeutic option for patients at this stage of disease.

## CONCLUSIONS

Clinical stage IV patients represent the most frequent group of GC who underwent any surgical procedures, and gastric bypass was the most common type of procedure performed. Jejunostomy was an independent factor associated with postoperative mortality and worse survival. Conversely, an improvement in survival was observed in patients who underwent resection. Accordingly, the identification of patients who would benefit from surgical resection may improve long-term survival in selected cases and avoid futile procedures.
